# Shelterin Component TPP1 Drives Tumor Progression and Predicts Poor Prognosis in Hepatocellular Carcinoma

**DOI:** 10.3390/biomedicines14020364

**Published:** 2026-02-04

**Authors:** Jung Eun Jang, Hye Seon Kim, Jin Seoub Kim, Jae Mo Han, Hee Sun Cho, Kwon Yong Tak, Ji Won Han, Pil Soo Sung, Si Hyun Bae, Jeong Won Jang

**Affiliations:** 1 The Catholic University Liver Research Center, The Catholic University of Korea, Seoul 06591, Republic of Korea; jsilver237@naver.com (J.E.J.); hseon7121@naver.com (H.S.K.); topiary@catholic.ac.kr (J.S.K.); kin41255@naver.com (J.M.H.); jhs-cho@hanmail.net (H.S.C.); eworldcupzps@naver.com (K.Y.T.); tmznjf@catholic.ac.kr (J.W.H.); pssung@catholic.ac.kr (P.S.S.); baesh@catholic.ac.kr (S.H.B.); 2 Cancer Research Institute, College of Medicine, The Catholic University of Korea, Seoul 06591, Republic of Korea; 3 Department of Medical Sciences, Graduate School of The Catholic University of Korea, Seoul 06591, Republic of Korea; 4 Department of Internal Medicine, College of Medicine, The Catholic University of Korea, Seoul 06591, Republic of Korea

**Keywords:** telomere, shelterin, prognostication, liver cancer

## Abstract

**Background/Objectives**: Telomere dysfunction and the shelterin complex are implicated in cancer, yet the specific functions and interactions of telomerase and shelterin genes in hepatocellular carcinoma (HCC) tumorigenesis remain poorly understood. This study aims to investigate the clinico-biological functions and collaborative contributions of telomerase and shelterin components in hepatocarcinogenesis. **Methods**: We analyzed tumor and matched non-tumor tissues from 274 HCC patients who underwent hepatectomy. Telomere-related parameters, including TERT (telomerase reverse transcriptase) expression and telomere length measured by qRT-PCR, telomerase activity assessed by the Telomerase Repeated Amplification Protocol assay, and six shelterin components analyzed by RNA sequencing, were correlated with clinicopathological features. siRNA-mediated knockdown of TPP1 (POT1–TIN2 organizing protein) was performed to evaluate its regulatory effect on TERT expression. Findings were externally validated. **Results**: TERT and TPP1 were upregulated in tumors with increased telomerase activity and shortened telomere length. Among the shelterin components, TPP1 showed the strongest correlation with TERT, and its expression increased with tumor multiplicity and advancing stage. TPP1 expression also correlated with proliferation-associated genes, consistent with Gene Set Enrichment Analysis suggesting TPP1 involvement in proliferative activity. TPP1 knockdown suppressed TERT protein expression and inhibited HCC cell proliferation, with the strongest anti-proliferative effect observed after dual TERT–TPP1 knockdown. Clinically, high TPP1 expression was associated with significantly earlier HCC recurrence, and co-high expression of TPP1–TERT was linked to significantly worse survival after hepatectomy. **Conclusions**: The TERT–TPP1 axis enhances proliferative activity and is associated with aggressive features and poor outcomes in HCC. TPP1 represents a potential therapeutic target and prognostic biomarker for HCC.

## 1. Introduction

Hepatocellular carcinoma (HCC) is one of the most common and lethal malignancies globally, often developing in patients with chronic liver disease and cirrhosis. Despite advancements in early detection and treatment options, the prognosis for HCC patients remains poor due to high rates of recurrence and limited long-term survival. The oncogenic mechanisms underlying HCC remain incompletely understood, which complicates effective therapeutic targeting and prognostication, ultimately leading to challenges in treatment and poor clinical outcomes.

Recently, there has been a growing focus on understanding the molecular biology of HCC, particularly the telomere dynamics and their involvement in tumor development. Research into telomere biology has provided new insights into the molecular mechanisms of HCC, particularly regarding telomerase activation and telomere maintenance [1,2,3]. Key molecular alterations, including mutations in the TERT promoter, have emerged as early events in hepatocarcinogenesis and are increasingly recognized as potential diagnostic and prognostic markers [4,5]. In addition, TERT amplification, translocation, and viral insertion also contribute to telomerase activation, which plays a critical role in the tumorigenesis of HCC [1,6,7,8,9].

The shelterin complex is a crucial protein assembly that protects telomeres, TTAGGG repetitive DNA sequences at the ends of chromosomes, by preventing recognition as DNA damage. Composed of six core components including the telomeric repeat-binding factors (TRF) 1 and TRF2, the TRF1-interacting protein 2 (TIN2), protection of telomeres 1 (POT1), the POT1–TIN2 organizing protein (TPP1), and repressor/activator protein 1 (RAP1), shelterin regulates telomere length and structure, in part by modulating access of telomerase, the enzyme responsible for telomere elongation [10]. While its primary role lies in telomere protection and maintenance, emerging evidence suggests that shelterin components may also play a role in tumorigenesis [11,12]. Dysregulation of shelterin can lead to telomere dysfunction and genomic instability, thereby promoting tumorigenesis [13]. Thus, shelterin plays a dual role in maintaining chromosomal integrity and potentially influencing carcinogenesis through altered telomere dynamics. Although shelterin’s role in cancer is increasingly recognized [10,11,14], its tumor-promoting effects in the context of HCC remain largely unexplored.

Therefore, this study aims to investigate the carcinogenic role of shelterin components in HCC by analyzing their clinico-pathologic associations as well as their relationships with TERT expression, telomerase activity, and telomere length. In addition, we conducted functional assays to determine whether shelterin components contribute to cellular tumorigenesis, and collectively evaluated their potential as novel biomarkers for tumor aggressiveness and clinical outcomes following hepatectomy in HCC.

## 2. Materials and Methods

### 2.1. Patients and Treatment

Between January 2017 and December 2024, a total of 274 patients with HCC were enrolled at The Catholic university of Korea in Seoul, Korea. Fresh tumor and adjacent non-tumor tissue specimens were collected during hepatectomy, immediately frozen in liquid nitrogen, and stored at −80 °C. The diagnosis of HCC was established in accordance with the regional guidelines [15]. HCC stage was assigned according to the modified Union for International Cancer Control (mUICC) criteria [15], and histological grading was conducted using the Edmondson–Steiner system [16]. This study received IRB approval (KC19SESI0195) from The Catholic University of Korea, and all participants provided written informed consent.

### 2.2. TERT and Shelterin mRNA Expression

Total RNA was extracted from frozen tissues using the QIAzol Lysis Reagent (Qiagen, Hilden, Germany) according to the manufacturer’s instructions. cDNA was synthesized with the High-Capacity cDNA Reverse Transcription Kit (Thermo Fisher Scientific, Waltham, MA, USA). Relative transcript levels were quantified by quantitative real-time PCR using TaqMan Universal Master Mix (cat. no. 4324018, Applied Biosystems, Foster City, CA, USA) on an ABI ViiA 7 Real-Time PCR System (Applied Biosystems). Target sequences for TERT and shelterin components were amplified using TaqMan Gene Expression Assays (TERT: Hs00972650_m1; TPP1: Assay ID Hs00368526_g1, Applied Biosystems). All RT-qPCR measurements were normalized to GAPDH (Hs03929097_g1; Applied Biosystems).

### 2.3. Telomerase Activity Assay

Telomerase activity was measured using the Telomeric Repeat Amplification Protocol (TRAP) assay with the TRAPeze^®^ RT Telomerase Detection Kit (Cat. No. S7710; Merck KGaA, Darmstadt, Germany). Frozen tissues were homogenized in 200 µL CHAPS lysis buffer, incubated on ice for 30 min, and then centrifuged at 12,000× *g* for 20 min. The concentration of the supernatant was determined using the Bradford assay (Bio-Rad Laboratories; Cat. No. 5000006). Heat-inactivated samples (85 °C for 10 min) served as negative controls. Real-time PCR was performed in a CFX96 Touch™ Real-Time PCR Detection System (Bio-Rad Laboratories, Hercules, CA, USA) containing 25 µL PCR MasterMix (5× TRAP RT mix, 2 U Titanium Taq, and 2 µL lysate) and all telomerase activity was quantified using a TSR8 standard curve.

### 2.4. Telomere Length Measurement

Telomere lengths were measured using the Absolute Human Telomere Length Quantification qPCR Assay Kit (Catalog No. 8918; ScienCell Research Laboratories, Carlsbad, CA, USA) as previously described [5]. Genomic DNA was extracted from tissue samples using the DNeasy Blood & Tissue Kit (Qiagen). Each qPCR reaction included the genomic DNA template, telomere-specific primers, and 2× qPCR Master Mix. A reference genomic DNA sample with a known telomere length was also included for standardization. All assays were performed using a CFX96 Touch™ Real-Time PCR Detection System (Bio-Rad Laboratories, Hercules, CA, USA).

### 2.5. RNA Sequencing and TCGA Data Analysis

For bulk RNA-seq analysis, total RNA was extracted from human tissues using the QIAzol Lysis Reagent (Qiagen, Hilden, Germany), according to the manufacturer’s instructions. RNA-seq libraries were sequenced on an Illumina NovaSeq platform. Fragments per kilobase of transcript per million mapped reads (FPKM) values were used for expression visualization and comparison. Differential gene expression analysis was performed in R using the DESeq2 package based on raw read counts. Gene set enrichment analysis (GSEA) was performed with genome-wide genes ranked according to differential expression statistics to identify enriched Kyoto Encyclopedia of Genes and Genomes (KEGG) pathways and Hallmark pathways using the clusterProfiler package in R. Publicly available RNA-seq data from The Cancer Genome Atlas (TCGA) hepatocellular carcinoma cohort were accessed to examine the expression levels of shelterin complex genes and TERT using the TCGAbiolinks package in R (version 4.5.1).

### 2.6. Cell Culture and siRNA Transfection

HCC cell lines SK-Hep1 and HepG2.2.15 were obtained from Dr. Yoon at Seoul St. Mary’s Hospital, Republic of Korea. The cells were cultured in Dulbecco’s Modified Eagle Medium (DMEM; HyClone, Logan, UT, USA), supplemented with 10% fetal bovine serum (FBS), 1% HEPES buffer, and 1% antibiotic–antimycotic solution. All cells were maintained at 37 °C in a humidified incubator with a 5% CO_2_ atmosphere.

Small interfering RNAs (siRNAs) targeting TERT (siRNA ID: s370) and TPP1 (ACD; siRNA ID: 140746), as well as a scrambled negative control siRNA, were purchased from Thermo Fisher Scientific (Silencer Select siRNA; Waltham, MA, USA). SK-Hep1 cells were transfected with 50 nM siRNA using Lipofectamine™ RNAiMAX reagent (Thermo Fisher Scientific, Waltham, MA, USA) at approximately 80% confluence, while HepG2.2.15 cells underwent reverse transfection method under identical conditions. After 48 h of incubation, the cells were harvested for subsequent analyses.

### 2.7. Cell Proliferation Assay

Cell viability was evaluated using a CCK-8 assay (Cell Counting Kit-8, Abcam, ab228554) according to the manufacturer’s protocol. Cells were seeded into a 96-well plate at a uniform density and incubated for 24, 48, 72, and 96 h. Subsequently, 10 µL of CCK-8 reagent was added to each well, and the cells were incubated for 2 h at 37 °C. The absorbance of each well was measured at 460 nm using a VersaMax microplate reader (Molecular Devices, San Jose, CA, USA).

### 2.8. Western Blot Analysis

For protein extraction, cells were lysed in PRO-PREP™ Protein Extraction Solution (iNtRON Biotechnology, Seongnam, Republic of Korea). The lysis buffer was supplemented with a protease inhibitor cocktail (Sigma-Aldrich, St. Louis, MO, USA), as well as phosphatase inhibitor cocktails 2 and 3 (Sigma-Aldrich). The extracted proteins were separated by SDS-PAGE and transferred to polyvinylidene fluoride membranes. The membranes were blocked with 5% skim milk and incubated with the following primary antibodies: β-actin antibody (A5441, Sigma-Aldrich, St. Louis, MO, USA), TERT antibody (ab32020, Abcam, Cambridge, UK), and TPP1 antibody (ab112050, Abcam, Cambridge, UK). Protein bands were visualized using Clarity™ Western ECL substrate (Thermo Fisher Scientific, Waltham, MA, USA).

### 2.9. Statistical Analysis

All data are presented as mean ± standard deviation or median (interquartile range), as appropriate. Continuous variables were compared using Student’s *t*-test or the Mann–Whitney U test, while categorical variables were compared using the chi-square test. Correlations between two continuous variables were assessed by Spearman’s rank correlation test. Survival curves for overall survival and time to recurrence were estimated using the Kaplan–Meier method and compared with the log-rank test. Prognostic factors for overall survival and time to recurrence were evaluated using univariate and multivariate Cox proportional hazards models. A two-tailed *p*-value < 0.05 was considered statistically significant. All analyses were performed using IBM SPSS Statistics 26.0 (IBM Corp., Armonk, NY, USA) and GraphPad Prism 9.0 (GraphPad Software, San Diego, CA, USA).

## 3. Results

### 3.1. Baseline Characteristics

This study analyzed a total of 396 liver tissue samples, which included 152 tumor-only samples and 122 paired tumor and adjacent non-tumor samples, obtained from 274 patients who underwent surgical resection. The mean age of the patients was 60.1 ± 10.2 years, with 203 patients (74.0%) being male. The primary etiologies of liver disease were hepatitis B virus infection (*n* = 176; 64.2%), followed by hepatitis C virus infection (*n* = 15; 5.5%) and non-viral causes (*n* = 83; 30.3%). The mean tumor size was 3.6 ± 3.2 cm, and 50 patients (20.8%) had multiple tumors. Most patients exhibited preserved liver function, with 233 (85.0%) classified as Child–Pugh class A. The baseline characteristics of the study population are summarized in Table 1.

### 3.2. Telomere Biology in HCC Versus Non-HCC

**Figure 1:**
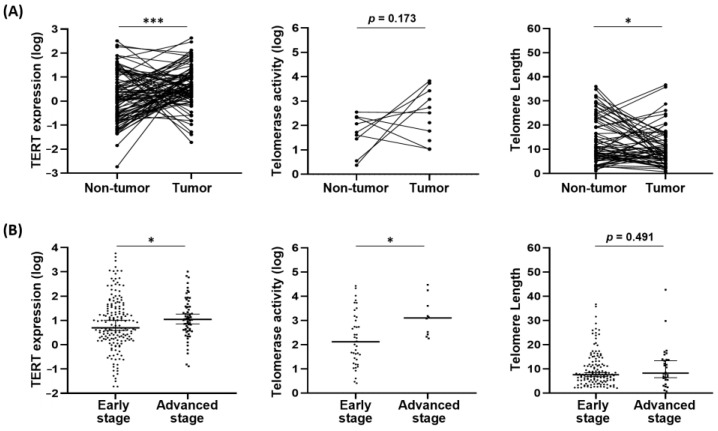
(**A**) Comparison of telomere biology parameters (TERT expression, telomerase activity, and telomere length) between paired tumor and non-tumor tissues. (**B**) Telomere biology parameters in tumor tissues according to tumor stage (stage I–II vs. III–IV). * *p* < 0.05, *** *p* < 0.001.

Telomere biology, including TERT expression, telomerase activity, and telomere length, was assessed in paired tumor and adjacent non-tumor tissue samples from 122 patients. TERT expression was significantly elevated in tumor tissues compared to their non-tumor counterparts. Tumors also showed a trend toward increased telomerase activity. In contrast, telomere length was notably shorter in tumor tissues than in non-tumor tissues, indicating enhanced telomere attrition in tumors (Figure 1A).

Within tumor samples, telomere biology appeared to correlate with tumor burden. Specifically, TERT expression and telomerase activity were significantly higher in advanced-stage tumors (mUICC stage III–IV) compared to early-stage HCC (mUICC stage I–II). Additionally, tumors from patients with advanced disease tended to have longer telomere length (Figure 1B). These findings suggest that telomere biology is aberrantly regulated and progressively activated as tumor stage and burden increase in HCC.

### 3.3. Shelterin Coordination and TPP1–TERT Association

**Figure 2:**
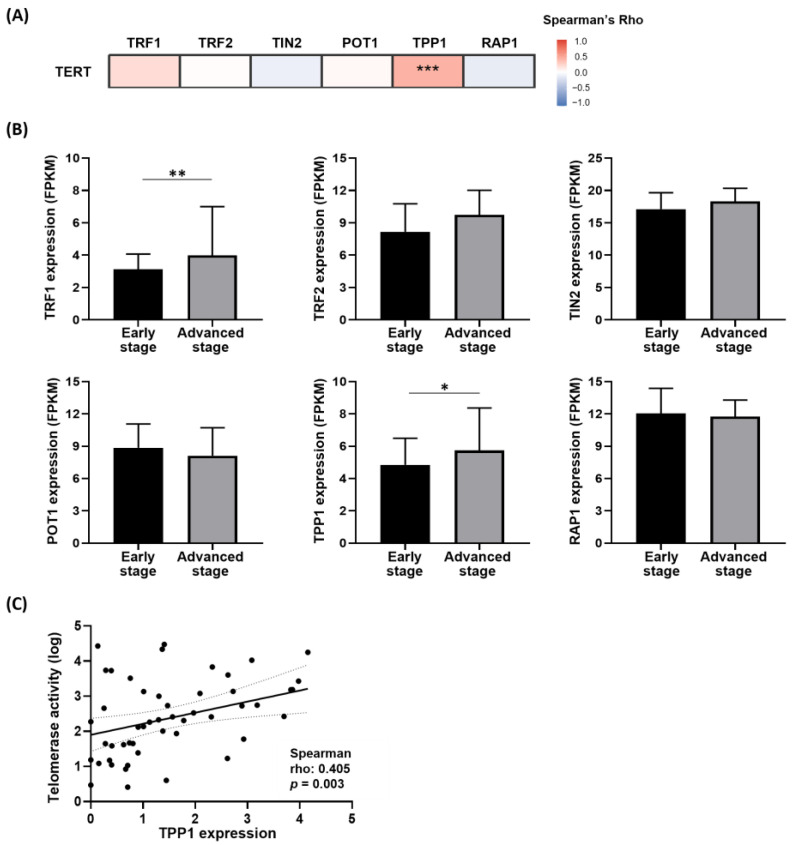
Shelterin components in HCC patients. (**A**) Heatmap of correlation between TERT expression and shelterin components (TRF1, TRF2, TIN2, POT1, TPP1, and RAP1) in tumor tissues based on RNA-sequencing data from our cohort. (**B**) Expression levels of the six shelterin components according to tumor stage (stage I–II vs. III–IV) in HCC tissues. (**C**) Correlation between TPP1 expression and telomerase activity (TRAP assay) in tumor tissues. * *p* < 0.05, ** *p* < 0.01, *** *p* < 0.001.

We investigated the potential involvement of the shelterin complex as a regulator of TERT in hepatocarcinogenesis using RNA-sequencing data from our patient cohort. Overall, all six components of the shelterin complex were positively correlated with one another. Notably, TPP1 exhibited the strongest correlation with TERT expression in tumor samples among these six components (Figure 2A). Additionally, TPP1 expression was significantly higher in advanced-stage HCC than in early-stage HCC (Figure 2B).

External validation using TCGA RNA-sequencing data revealed consistent results. Among the six shelterin components, TPP1 displayed the strongest positive correlation with TERT, and its expression was significantly higher in advanced-stage HCC than in early-stage HCC (Figure S1). Furthermore, in a subset of patients (*n* = 51) who underwent TRAP assays, TPP1 showed the strongest positive correlation with telomerase activity (Figure 2C).

Collectively, these findings suggest that TPP1 may play a central role among the shelterin components and is closely associated with telomere dysfunction in promoting hepatocarcinogenesis. Consequently, TPP1 was selected for further functional analyses.

### 3.4. Clinicopathological Features Associated with TPP1 in HCC

**Figure 3:**
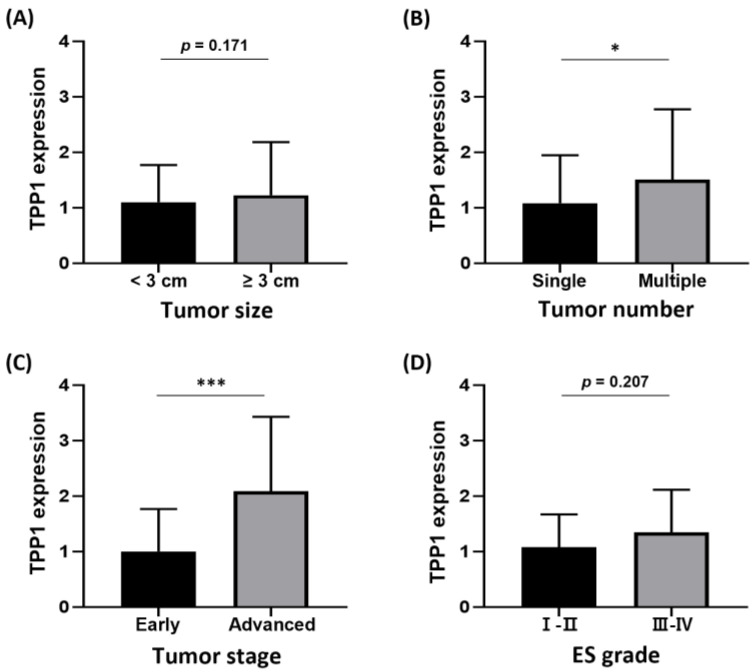
TPP1 expression according to clinicopathological features in HCC patients. qRT-PCR analysis of TPP1 mRNA levels according to (**A**) tumor size, (**B**) tumor multiplicity, (**C**) tumor stage, and (**D**) Edmondson–Steiner (ES) pathological grade. * *p* < 0.05, *** *p* < 0.001.

We analyzed the clinicopathological features associated with TPP1 expression to evaluate its potential as a clinically relevant shelterin biomarker for HCC. Elevated TPP1 expression was significantly linked to tumor multiplicity and more advanced tumor stages compared to tumors without these characteristics (Figure 3). Additionally, higher TPP1 levels were marginally associated with poorer pathological differentiation and were more frequently observed in patients with larger tumor sizes, supporting an association between TPP1 expression and aggressive tumor features in HCC.

### 3.5. Involvement of TPP1 in Oncogenic Pathways

**Figure 4:**
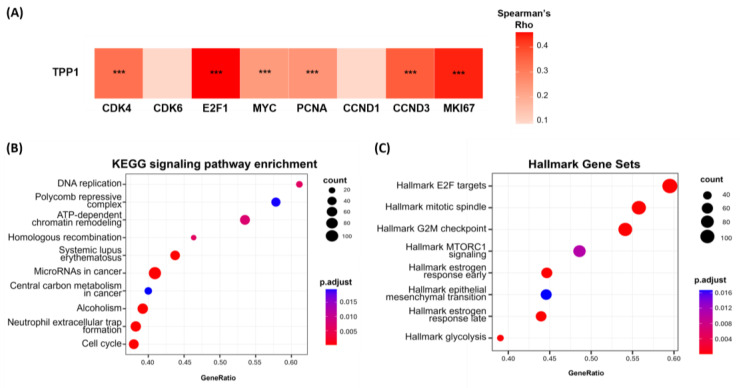
Tumorigenic effects of TPP1 in HCC. (**A**) TPP1 expression positively correlates with proliferation-associated genes, including CDK4, CDK6, E2F1, MYC, PCNA, CCND1, CCND3, and MKI67, in HCC. (**B**,**C**) GSEA dot plot illustrating the top 10 enriched pathways from KEGG and Hallmark gene set analyses, ranked by gene ratio. *** *p* < 0.001.

The tumorigenic role of TPP1 was examined by correlating its expression with various oncogenic signaling pathways using RNA-sequencing data from our cohort. We found that TPP1 expression was significantly associated (*p* < 0.05) with several tumor-promoting genes, including CDK4, E2F1, MYC, PCNA, CCND3, and MKI67 (Figure 4A).

To further explore the biological pathways linked to TPP1, we conducted a KEGG pathway analysis comparing the TPP1-high and TPP1-low groups. The analysis revealed significant enrichment in pathways related to cell proliferation, such as DNA replication, Polycomb repressive complex, ATP-dependent chromatin remodeling, homologous recombination, and the cell cycle (Figure 4B). In addition, hallmark gene set analysis corroborated these findings, indicating a notable upregulation of E2F targets, mitotic spindle, G2M checkpoint, and mTORC1 signaling in the TPP1-high group (Figure 4C). Overall, these results suggest that TPP1 plays a functional role in cell-cycle progression and proliferative signaling in HCC.

### 3.6. Tumorigenic Effects of TPP1

**Figure 5:**
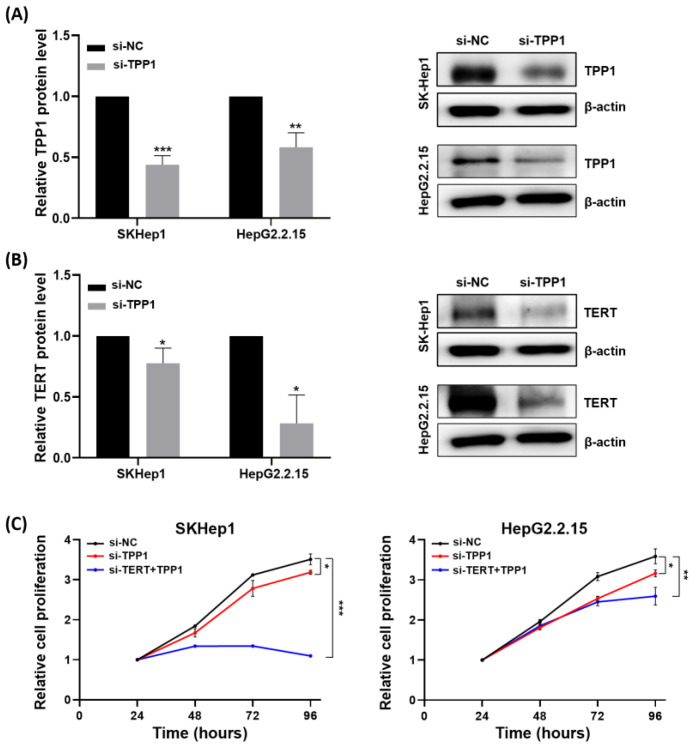
Effects of TPP1 on cell proliferation in HCC cell lines. (**A**) TPP1 protein expression was reduced following si-TPP1 transfection, as determined by Western blot analysis in SK-Hep1 and HepG2.2.15 cells. (**B**) TERT protein expression was also decreased after si-TPP1 transfection, as measured by Western blot analysis in SK-Hep1 and HepG2.2.15 cells. (**C**) Cell viability assays following TPP1 and TERT knockdown. Dual knockdown of TPP1 and TERT resulted in the strongest inhibition of cell proliferation compared with single knockdown or control groups in SK-Hep1 and HepG2.2.15 cells. * *p* < 0.05, ** *p* < 0.01, *** *p* < 0.001.

To further investigate the oncogenic role of TPP1, TPP1-targeting siRNA was transfected into human hepatoma cell lines, including SK-Hep1 and HepG2.2.15. Following si-TPP1 transfection, TPP1 protein levels were significantly downregulated in both SK-Hep1 and HepG2.2.15 cells, as shown in Figure 5A. Notably, inhibition of TPP1 also resulted in a significant reduction in TERT protein levels (Figure 5B), suggesting that TPP1 may partially regulate TERT expression. Functional analysis using the CCK-8 assay revealed that silencing TPP1 significantly suppressed the proliferative capacity of both SK-Hep1 and HepG2.2.15 cells. Interestingly, simultaneous silencing of both TPP1 and TERT resulted in the most pronounced inhibition of cell proliferation in these cell lines (Figure 5C), suggesting that the coordinated activity of TPP1 and TERT promotes tumorigenesis, at least in part, by regulating cancer cell proliferation.

### 3.7. Clinical Outcomes According to TPP1 Expression

Given the cancer-promoting role of the shelterin component TPP1, we evaluated clinical outcomes according to TPP1 expression levels. During a median follow-up period of 24 months (range: 0.2–60.00), 98 patients (42.1%) experienced tumor recurrence following hepatectomy, and 46 patients (17.6%) died.

**Figure 6:**
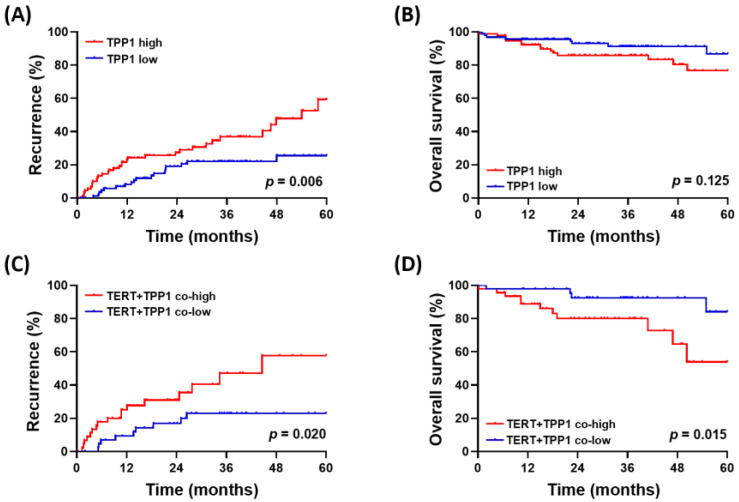
Clinical outcomes according to TPP1 expression and its relation to TERT expression. (**A**) Time to recurrence and (**B**) overall survival according to TPP1 expression. (**C**) Time to recurrence and (**D**) overall survival according to the co-expression patterns of TPP1 and TERT.

Patients with high TPP1 expression experienced recurrence significantly earlier than those with low expression (*p* = 0.006; Figure 6A). In survival analysis, patients with high TPP1 expression exhibited a trend toward poorer overall survival, although this difference did not reach statistical significance (*p* = 0.125; Figure 6B). In the multivariable analysis, high TPP1 expression was independently associated with postoperative recurrence in HCC (HR = 2.072, 95% CI: 1.147–3.742; *p* = 0.016; Table S1). Notably, when considering both TPP1 and TERT expression levels, patients with co-high TPP1–TERT expression exhibited significantly higher recurrence (*p* = 0.020) rates as well as poorer overall survival (*p* = 0.015) compared to those with co-low expression (Figure 6C,D). In the multivariable analysis, co-high TPP1–TERT expression emerged as an independent predictor of increased postoperative recurrence and poorer overall survival in HCC (Table 2).

Consistent with these findings, an exploratory analysis of the TCGA dataset revealed that patients with high TPP1 expression tended to experience earlier recurrence compared to those with low expression (*p* = 0.017; Figure S2A). The TCGA dataset also showed significantly worse overall survival outcomes in patients with high TPP1 expression (*p* < 0.001; Figure S2B), further supporting the potential role of TPP1 as a negative prognostic biomarker in HCC.

## 4. Discussion

This study investigated the role of the shelterin complex in liver carcinogenesis. Among the six known components of the shelterin complex that regulate telomerase activity [11], TPP1 exhibited the strongest association with telomere dysfunction. It was significantly overexpressed in tumor tissues compared to adjacent non-tumor tissues and showed a strong correlation with tumor TERT expression and telomerase activity. Additionally, TPP1 expression was linked to aggressive tumor characteristics, including tumor multiplicity and advanced stages of HCC. Elevated levels of TPP1 were associated with earlier recurrence following hepatectomy, and co-high expression of TPP1 and TERT was correlated with both recurrence and overall survival. These findings suggest that TPP1 may contribute to HCC progression, in part, by regulating TERT expression.

First of all, our study confirmed the abnormal functions of the TERT axis in hepatocarcinogenesis [1,5]. Tumors showed higher TERT expression and telomerase activity, but shorter telomere length compared to non-tumors, reflecting cumulative cellular damage and regeneration during chronic liver disease. In the early phase of tumor development, repeated cell division and genomic stress drive progressive telomere attrition, and telomerase activity remains insufficient to elongate all telomeres, resulting in continued bulk telomere shortening despite preferential elongation of the shortest telomeres. As tumor stage progresses, increasing TERT activity reaches levels sufficient to stabilize or elongate telomeres [2,17], leading to relatively longer telomere length in advanced-stage HCC [5]. This dynamic process facilitates the clonal expansion of genetically unstable cells and represents a key step in hepatocarcinogenesis [18].

Among the shelterin components, TPP1 showed the strongest association with TERT, highlighting its role in TERT-related oncogenic activity. TPP1 expression correlated with TERT levels, increased tumor number, poor differentiation, and advanced tumor stage, suggesting a role in aggressive tumor behavior via the telomere/telomerase pathway. Similar associations between TPP1 and telomerase activity have been observed in other malignancies, including colorectal cancer [19] and cervical cancer [20], suggesting a broader oncogenic role of TPP1 across cancer types. TPP1 may be particularly important in hepatocarcinogenesis due to its dominant role in telomerase recruitment, especially in HCC developing in chronic inflammation and cirrhosis, where telomere attrition and hepatocyte turnover drive telomerase activation [1,21]. Indeed, earlier work has reported a positive correlation between TPP1 and hTERT expression in HCC, supporting its role in telomere maintenance during hepatocarcinogenesis [12]. However, since different shelterin components are linked to specific tumor types [10,11,14], further studies are needed to determine whether the correlations between specific shelterin proteins and TERT abnormalities are dependent on cancer type or context.

The tumorigenic significance of TPP1 was further supported by its association with post-resection outcomes in our analysis. Patients exhibiting high TPP1 expression experienced significantly earlier recurrence following hepatectomy. Moreover, TPP1 was identified as an independent predictor of recurrence after HCC resection. The lack of significance for overall survival by TPP1 levels may reflect limited survival events due to recent patient enrollment in our cohort and concurrent advances in post-recurrence treatment strategies. Nevertheless, TCGA data demonstrated that high TPP1 expression was significantly associated with both recurrence and poor overall survival. While telomere dysfunction has consistently been linked to poor prognosis in HCC [5,22,23], our findings emphasize that, beyond the direct oncogenic effects of TERT alterations, TPP1 as a shelterin component also holds prognostic significance in this disease.

The role of TPP1 in HCC tumorigenesis remains incompletely understood. TPP1 maintains telomere integrity by recruiting telomerase and enhancing its activity, thereby promoting telomere elongation and enabling sustained cellular proliferation [18,24]. It also protects telomere ends from DNA damage responses that induce senescence or apoptosis, thus maintaining chromosomal stability [25]. Dysregulation or overexpression of TPP1 may contribute to tumorigenesis [26]. In our exploratory analyses using RNA-seq data, we found a significant correlation between TPP1 expression and the enrichment of genes and pathways associated with cell proliferation. Similar to its reported role in other cancers [20,26], these findings suggest that TPP1 may confer malignant potential to HCC by enhancing proliferative capacity. Consistent with the transcriptomic results, siRNA-mediated knockdown of TPP1 in vitro suppressed TERT protein expression, resulting in reduced cell proliferation. Patients in our clinical cohort exhibiting high TPP1-TERT co-expression experienced significantly earlier recurrence and worse overall survival. Together, these data suggest that TPP1 may contribute to hepatocarcinogenesis, at least in part, by functionally cooperating with TERT to enhance TERT-driven oncogenic processes, particularly in telomerase-active cancers such as HCC.

From a therapeutic standpoint, the feasibility of small-molecule or peptide-based inhibitors targeting TPP1–TERT [27] or TPP1–POT1 [28] interactions warrants further exploration as a strategy to selectively disrupt telomerase recruitment or processivity in HCC. However, given the essential role of TPP1 in telomere maintenance in normal stem cells, such approaches would require high specificity and careful evaluation of on-target toxicity.

This study has several limitations. Its retrospective, single-center design and exclusive inclusion of surgically treated HCC patients may have introduced selection bias and limited generalizability, particularly to patients with non-surgical disease. The observed associations may therefore preferentially reflect tumor biology in resectable HCC, underscoring the need for future multi-center and prospective studies for validation. Although the overall cohort size was adequate for exploratory analyses, limited tissue availability restricted the feasibility of comprehensive tissue-based assays, including TRAP and other TERT-related functional studies, thereby reducing statistical power in these sub-analyses. These results should thus be interpreted with caution. In addition, while the proposed regulatory link between TPP1 and TERT is supported by correlative analyses and knockdown experiments, it remains observational, and future studies incorporating rescue or promoter-based assays will be required to establish causality. Nevertheless, our integrated analysis of key components of telomere biology, supported by external datasets and functional assays, provides mechanistic insight into the role of TPP1 in HCC.

## 5. Conclusions

In conclusion, this study identifies TPP1 as a pivotal shelterin component that promotes HCC progression by enhancing proliferative capacity and associating with aggressive clinical outcomes. Our findings further suggest that TPP1 may cooperate with TERT to potentiate telomerase-driven oncogenic processes in HCC. These results highlight the potential of TPP1 as both a prognostic biomarker and a therapeutic target in HCC. Our findings provide a basis for future studies to delineate the mechanisms of TPP1-mediated tumorigenesis and to explore strategies for targeting telomere regulation in liver cancer.

## Figures and Tables

**Table 1:** Baseline characteristics of the study population.

Characteristics	HCC Group (*n* = 274)
Sex	
Male	203 (74.0)
Female	71 (26.0)
Age (years)	60.1 ± 10.2
Cause of liver disease	
HBV	176 (64.2)
HCV	15 (5.5)
Non-viral	83 (30.3)
AST (U/L)	38.0 (14.0–912.0)
ALT (U/L)	32.0 (6.0–849.0)
AFP (ng/mL)	11.66 (0.9–60,500.0)
Child–Pugh class	
A	233 (85.0)
B + C	41 (15.0)
Tumor size (cm)	3.6 ± 3.2
Tumor number	
Single	205 (79.2)
Multiple	50 (20.8)
PVT	
Presence	40 (15.7)
Absence	215 (84.3)
mUICC stage	
I–II	202 (71.9)
III–IV	72 (25.3)
Edmondson–Steiner grade	
1–2	63 (34.6)
3–4	119 (65.4)

HBV, hepatitis B virus; HCV, hepatitis C virus; AST, aspartate aminotransferase; ALT, alanine aminotransferase; AFP, alpha-fetoprotein; PVT, portal vein thrombosis; mUICC, modified Union for International Cancer Control; Data are expressed as mean ± SD or median (interquartile range). Figures in parentheses indicate percentage.

**Table 2:** Factors for clinical outcomes.

Variables	Overall Survival			Recurrence		
	Univariate	Multivariate		Univariate	Multivariate	
	*p*	HR (95% CI)	*p*	*p*	HR (95% CI)	*p*
Male sex	0.056			0.328		
Age > 60 years	0.119			0.003	0.513 (0.287–0.917)	0.513
Cause of liver disease	0.651			0.636		
AST ≥ 40 U/L	0.026	1.737 (0.602–5.015)	0.307	0.001	3.336 (1.524–7.035)	0.003
ALT ≥ 40 U/L	0.219			0.047	0.638 (0.294–1.385)	0.258
Tumor size ≥ 3.6 cm	0.001	0.716 (0.264–1.941)	0.511	0.000	1.991 (1.095–3.619)	0.024
Tumor multiplicity	0.000	3.349 (1.203–9.320)	0.021	0.000	1.827 (0.885–3.771)	0.103
α-fetoprotein ≥ 10 ng/mL	0.017	4.326 (1.289–14.519)	0.018	0.001	2.405 (1.349–4.286)	0.003
Edmondson grade	0.015	5.447 (0.673–44.062)	0.112	0.078		
Child–Pugh Class (A/B + C)	0.026	3.849 (0.941–15.749)	0.061	0.426		
Co-high TPP1-TERT	0.015	5.997 (2.103–17.098)	0.001	0.020	2.794 (1.464–5.335)	0.002

HR, hazard ratio; CI, confidence interval; AST, aspartate aminotransferase; ALT, alanine aminotransferase; TPP1, telomeric protection protein 1; TERT, telomerase reverse transcriptase.

## Data Availability

The data presented in this study are available on request from the corresponding author. Associated clinical data cannot be provided to maintain patient confidentiality.

## Supplementary Materials

The following supporting information can be downloaded at: https://www.mdpi.com/article/10.3390/biomedicines14020364/s1, Figure S1: Coordination of shelterin components and strong association between TPP1 and TERT in HCC the TCGA-LIHC cohort; Figure S2: Prognostic impact of TPP1 expression in the TCGA-LIHC cohort; Table S1: Univariate and multivariate analyses of clinical outcomes.

## Abbreviations

The following abbreviations are used in this manuscript:

HCC	Hepatocellular carcinoma
TERT	Telomerase reverse transcriptase
TRF	Telomeric repeat-binding factors
TIN2	TRF1-interacting protein 2
POT1	Protection of telomeres 1
TPP1	POT1–TIN2 organizing protein
RAP1	Repressor/activator protein 1
TRAP	Telomeric Repeat Amplification Protocol
PCR	Polymerase chain reaction
siRNA	Small Interfering RNA
TCGA	The Cancer Genome Atlas
GSEA	Gene Set Enrichment Analysis
KEGG	Kyoto Encyclopedia of Genes and Genomes

## References

[1] Nault J.C., Ningarhari M., Rebouissou S., Zucman-Rossi J. (2019). The role of telomeres and telomerase in cirrhosis and liver cancer. Nat. Rev. Gastroenterol. Hepatol..

[2] In der Stroth L., Tharehalli U., Gunes C., Lechel A. (2020). Telomeres and Telomerase in the Development of Liver Cancer. Cancers.

[3] Ma Z.X., Yang C.M., Lee M.G., Tu H. (2019). Telomerase reverse transcriptase promoter mutations in hepatocellular carcinogenesis. Hepatoma Res..

[4] Nault J.C., Calderaro J., Di Tommaso L., Balabaud C., Zafrani E.S., Bioulac-Sage P., Roncalli M., Zucman-Rossi J. (2014). Telomerase reverse transcriptase promoter mutation is an early somatic genetic alteration in the transformation of premalignant nodules in hepatocellular carcinoma on cirrhosis. Hepatology.

[5] Jang J.W., Kim J.S., Kim H.S., Tak K.Y., Lee S.K., Nam H.C., Sung P.S., Kim C.M., Park J.Y., Bae S.H. (2021). Significance of TERT Genetic Alterations and Telomere Length in Hepatocellular Carcinoma. Cancers.

[6] Totoki Y., Tatsuno K., Covington K.R., Ueda H., Creighton C.J., Kato M., Tsuji S., Donehower L.A., Slagle B.L., Nakamura H. (2014). Trans-ancestry mutational landscape of hepatocellular carcinoma genomes. Nat. Genet..

[7] Li C.L., Hsu C.L., Lin Y.Y., Ho M.C., Hu R.H., Chen C.L., Ho T.C., Lin Y.F., Tsai S.F., Tzeng S.T. (2024). HBV DNA Integration into Telomerase or MLL4 Genes and TERT Promoter Point Mutation as Three Independent Signatures in Subgrouping HBV-Related HCC with Distinct Features. Liver Cancer.

[8] Jang J.W., Kim H.S., Kim J.S., Lee S.K., Han J.W., Sung P.S., Bae S.H., Choi J.Y., Yoon S.K., Han D.J. (2021). Distinct Patterns of HBV Integration and TERT Alterations between in Tumor and Non-Tumor Tissue in Patients with Hepatocellular Carcinoma. Int. J. Mol. Sci..

[9] Kim J.S., Kim H.S., Tak K.Y., Han J.W., Nam H., Sung P.S., Lee S.W., Kwon J.H., Bae S.H., Choi J.Y. (2025). Male preference for TERT alterations and HBV integration in young-age HBV-related HCC: Implications for sex disparity. Clin. Mol. Hepatol..

[10] Patel T.N., Vasan R., Gupta D., Patel J., Trivedi M. (2015). Shelterin proteins and cancer. Asian Pac. J. Cancer Prev..

[11] Chakraborty S., Banerjee S. (2024). Combatting cellular immortality in cancers by targeting the shelterin protein complex. Biol. Direct.

[12] Kim H., Yoo J.E., Cho J.Y., Oh B.K., Yoon Y.S., Han H.S., Lee H.S., Jang J.J., Jeong S.H., Kim J.W. (2013). Telomere length, TERT and shelterin complex proteins in hepatocellular carcinomas expressing “stemness”-related markers. J. Hepatol..

[13] Brankiewicz-Kopcinska W., Kallingal A., Krzemieniecki R., Baginski M. (2024). Targeting shelterin proteins for cancer therapy. Drug Discov. Today.

[14] Cacchione S., Biroccio A., Rizzo A. (2019). Emerging roles of telomeric chromatin alterations in cancer. J. Exp. Clin. Cancer Res..

[15] Korean Liver Cancer Assotiation, National Cancer Center Korea (2023). 2022 KLCA-NCC Korea practice guidelines for the management of hepatocellular carcinoma. J. Liver Cancer.

[16] Edmondson H.A., Steiner P.E. (1954). Primary carcinoma of the liver: A study of 100 cases among 48,900 necropsies. Cancer.

[17] Okamoto K., Seimiya H. (2019). Revisiting Telomere Shortening in Cancer. Cells.

[18] Bernal A., Tusell L. (2018). Telomeres: Implications for Cancer Development. Int. J. Mol. Sci..

[19] Witkowska A., Gumprecht J., Glogowska-Ligus J., Wystrychowski G., Owczarek A., Stachowicz M., Bocianowska A., Nowakowska-Zajdel E., Mazurek U. (2010). Expression profile of significant immortalization genes in colon cancer. Int. J. Mol. Med..

[20] Wang Q.L., Gong C., Meng X.Y., Fu M., Yang H., Zhou F., Wu Q., Zhou Y. (2024). TPP1 is associated with risk of advanced precursors and cervical cancer survival. PLoS ONE.

[21] Oh B.K., Jo Chae K., Park C., Kim K., Jung Lee W., Han K.H., Nyun Park Y. (2003). Telomere shortening and telomerase reactivation in dysplastic nodules of human hepatocarcinogenesis. J. Hepatol..

[22] Lee H.W., Park T.I., Jang S.Y., Park S.Y., Park W.J., Jung S.J., Lee J.H. (2017). Clinicopathological characteristics of TERT promoter mutation and telomere length in hepatocellular carcinoma. Medicine.

[23] Ningarhari M., Caruso S., Hirsch T.Z., Bayard Q., Franconi A., Vedie A.L., Noblet B., Blanc J.F., Amaddeo G., Ganne N. (2021). Telomere length is key to hepatocellular carcinoma diversity and telomerase addiction is an actionable therapeutic target. J. Hepatol..

[24] Zhong F.L., Batista L.F., Freund A., Pech M.F., Venteicher A.S., Artandi S.E. (2012). TPP1 OB-fold domain controls telomere maintenance by recruiting telomerase to chromosome ends. Cell.

[25] Tejera A.M., Stagno d’Alcontres M., Thanasoula M., Marion R.M., Martinez P., Liao C., Flores J.M., Tarsounas M., Blasco M.A. (2010). TPP1 is required for TERT recruitment, telomere elongation during nuclear reprogramming, and normal skin development in mice. Dev. Cell.

[26] Wen J., Zhong X., Gao C., Yang M., Tang M., Yuan Z., Wang Q., Xu L., Ma Q., Guo X. (2023). TPP1 Inhibits DNA Damage Response and Chemosensitivity in Esophageal Cancer. Crit. Rev. Eukaryot. Gene Expr..

[27] Guterres A.N., Villanueva J. (2020). Targeting telomerase for cancer therapy. Oncogene.

[28] Welfer G.A., Freudenthal B.D. (2023). Recent advancements in the structural biology of human telomerase and their implications for improved design of cancer therapeutics. NAR Cancer.

