# Supplementary material for: Shelterin Component TPP1 Drives Tumor Progression and Predicts Poor Prognosis in Hepatocellular Carcinoma
Source: Biomedicines. 2026 Feb 4;14(2):364. doi: 10.3390/biomedicines14020364 (PMC12938003; doi:10.3390/biomedicines14020364)
Supplement: Supplementary file 1 [file biomedicines-14-00364-s001.zip › biomedicines-4100517-supplementary.pdf]

*Supplementary Materials*

# Shelterin component TPP1 drives tumor progression and predicts poor prognosis in hepatocellular carcinoma

**Jung Eun Jang[1,2,3] , Hye Seon Kim[1,2,3], Jin Seoub Kim[1,2,3] , Jae Mo Han[1,2,3], Hee Sun Cho[1,4,] , Kwon Yong Tak[1,4], Ji Won Han[1,4], Pil Soo Sung[1,4], Si Hyun Bae[1,4], Jeong Won Jang[1,2,4]***

[1] The Catholic University Liver Research Center, The Catholic University of Korea;
[2] Cancer Research Institute, College of Medicine, The Catholic University of Korea;
[3] Department of Medical Sciences, Graduate School of The Catholic University of Korea;
[4] Department of Internal Medicine, College of Medicine, The Catholic University of Korea, Seoul, Korea

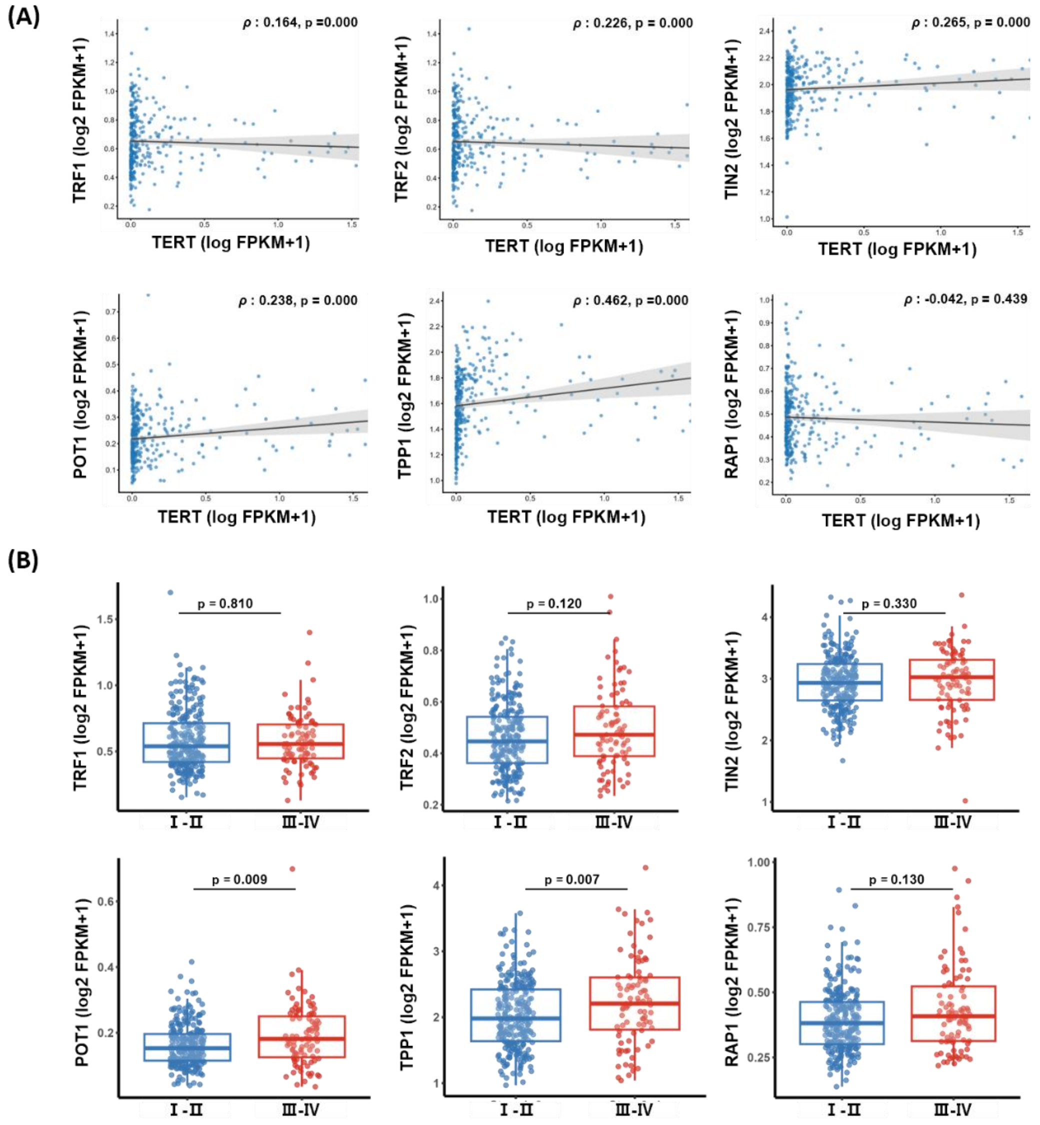


**Figure S1.** Coordination of shelterin components and strong association between TPP1 and TERT in HCC the TCGA-LIHC cohort. (A) Spearman correlation analysis between TERT and shelteirn components in TCGA-LIHC tumor samples. (B) Comparison of shelterin expression in RNA-seq data from TCGA-LIHC between AJCC stage I–II and stage III–IV tumors. TERT, telomerase reverse transcriptase; HCC, hepatocellular carcinoma; TRF, telomeric repeat-binding factors; POT1, protection of telomeres 1; TPP1, POT1-TIN2 organizing protein; TIN2, TRF1 and TRF2 interacting nuclear protein 2; RAP1, repressor/activator protein 1.

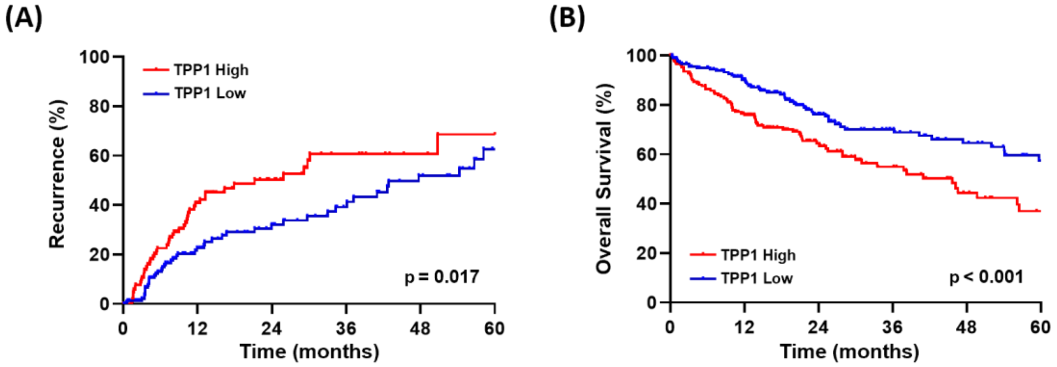


**Figure S2.** Prognostic impact of TPP1 expression in the TCGA-LIHC cohort. (A-B) Kaplan-Meier Recurrence-free survival and overall survival by TPP1 expression in TCGA dataset

**Table S1.** Univariate and multivariate analyses of clinical outcomes

| Variables | Overall Survival | | | Recurrence | | |
|---|---|---|---|---|---|---|
| | Univariate | Multivariate | | Univariate | Multivariate | |
| | *p* | HR (95% CI) | *p* | *p* | HR (95% CI) | *p* |
| Male sex | 0.056 | | | 0.328 | | |
| Age > 60 years | 0.119 | | | 0.003 | 0.534 (0.300–0.950) | 0.033 |
| Cause of liver disease | 0.651 | | | 0.636 | | |
| AST ≥ 40 U/L | 0.026 | 1.995 (0.836–4.763) | 0.082 | 0.001 | 2.432 (1.165–5.076) | 0.018 |
| ALT ≥ 40 U/L | 0.092 | | | 0.047 | 0.821 (0.382–1.762) | 0.612 |
| Tumor size ≥ 3.6 cm | 0.001 | 1.055 (0.442–2.522) | 0.904 | 0.000 | 2.123 (1.170–3.852) | 0.013 |
| Tumor multiplicity | 0.000 | 5.747 (2.478–13.279) | 0.000 | 0.000 | 1.821 (0.865–3.830) | 0.114 |
| $\alpha$-fetoprotein ≥ 10 ng/mL | 0.017 | 2.462 (0.950–6.381) | 0.087 | 0.001 | 2.155 (1.223–3.798) | 0.008 |
| Edmondson grade | 0.015 | 3.379 (0.750–15.223) | 0.087 | 0.078 | | |
| Child-Pugh Class (A/B+C) | 0.026 | 1.973 (0.644–6.049) | 0.082 | 0.426 | | |
| High TPP1 | 0.126 | | | 0.035 | 2.072 (1.147–3.742) | 0.016 |
| High TERT | 0.627 | | | 0.355 | | |
